# Menaquinone-7 Supplementation Increases Multiple Advanced Glycation End-Products and Oxidation Markers in Zucker Diabetic Fatty Rats

**DOI:** 10.3390/nu17172733

**Published:** 2025-08-23

**Authors:** Ingo Mrosewski, Thomas Fleming, Gundula Schulze-Tanzil, Christian Werner, Clemens Gögele, Valeriya Mantel, Maria Kokozidou, Thomas Bertsch

**Affiliations:** 1 MVZ MDI Limbach Berlin, Aroser Allee 84, 13407 Berlin, Germany; valeria_mantel@yahoo.de; 2 Department for Endocrinology, Diabetology, Metabolic Diseases and Clinical Chemistry, University Hospital Heidelberg, Im Neuenheimer Feld 410, 69120 Heidelberg, Germany; thomas.fleming@med.uni-heidelberg.de; 3 German Center for Diabetes Research (DZD), Helmholtz Center Munich, Ingolstädter Landstrasse 1, 85764 Neuherberg, Germany; 4 Joint Heidelberg-IDC Translational Diabetes Program, Internal Medicine I, Heidelberg University Hospital, Im Neuenheimer Feld 410, 69120 Heidelberg, Germany; 5 Institute of Anatomy and Cell Biology, Paracelsus Medical University, Nuremberg and Salzburg, Prof. Ernst Nathan Str. 1, 90419 Nuremberg, Germany; gundula.schulze@pmu.ac.at (G.S.-T.); christian.werner@pmu.ac.at (C.W.); clemens.goegele@pmu.ac.at (C.G.); maria.kokozidou@pmu.ac.at (M.K.); 6 Department V—Life Sciences and Technology, Berlin University of Applied Sciences and Technology, Luxemburger Str. 10, 13353 Berlin, Germany; 7 Institute of Clinical Chemistry, Laboratory Medicine and Transfusion Medicine, Nuremberg General Hospital, Paracelsus Medical University, Prof. Ernst Nathan Str. 1, 90419 Nuremberg, Germany; thomas.bertsch@klinikum-nuernberg.de

**Keywords:** type 2 diabetes mellitus, dicarbonyls, advanced glycation end-products, nitration adducts, oxidation adducts, Zucker diabetic fatty (ZDF) rat, menaquinone-7 (MK-7)

## Abstract

**Background:** Dicarbonyls and advanced glycation end-products (AGEs) contribute to oxidative stress, inflammation, and complications in type 2 diabetes mellitus (T2DM). Menaquinone-7 (MK-7), a vitamin K2 subtype, has shown benefits for glucose tolerance and vascular health in some studies. We evaluated the impact of MK-7 on dicarbonyls, free AGEs, and protein nitration/oxidation adducts in a rat model of T2DM. **Methods:** Male heterozygous (fa/+, control) and homozygous (fa/fa, diabetic) Zucker Diabetic Fatty rats were fed a diabetogenic diet without or with MK-7 for 12 weeks. After sacrifice, plasma dicarbonyls as well as plasma and urinary levels of free AGEs and protein nitration/oxidation adducts were quantified by isotope dilution tandem mass spectrometry. **Results:** Diabetic rats showed significantly increased plasma glyoxal, 3-deoxyglucosone, and fructosyl-lysine with non-significant trends toward increased methylglyoxal-derived hydroimidazolone and methionine sulfoxide, as well as reductions in methylglyoxal and dityrosine. Urinary carboxyethyl-lysine, carboxymethyl-lysine, fructosyl-lysine (all significant), and dityrosine (non-significant) were elevated in diabetic rats; glucosepane (non-significant) was reduced. MK-7 supplementation reduced no measured parameter but was associated with non-significant further increases in plasma glyoxal-derived hydroimidazolone, carboxyethyl-lysine, carboxymethyl-lysine, fructosyl-lysine, 3-nitrotyrosine, and methionine sulfoxide, as well as in urinary glyoxal-derived hydroimidazolone, carboxyethyl-lysine, fructosyl-lysine, and 3-nitrotyrosine, in diabetic rats. Correlation analysis revealed significant associations between glucose, dicarbonyls, AGEs, and oxidative markers. **Conclusions**: High-dose MK-7 supplementation did not improve dicarbonyl stress, AGE burden, or protein nitration/oxidation. With respect to available scientific evidence and our observations, the combination of glycemia-driven amplification of glycation and oxidative stress, as well as MK-7-induced glutathione depletion, were likely causative.

## 1. Introduction

Type 2 diabetes mellitus (T2DM), which accounts for approximately 90% of all diabetes cases, is characterized by chronic low-level inflammation and the accumulation of reactive metabolites such as dicarbonyls, advanced glycation end products (AGEs), and nitration and oxidation adducts [1,2,3,4,5,6,7,8].

These reactive species and their byproducts disrupt cellular signaling, promote apoptosis, and contribute to insulin resistance, pancreatic β-cell dysfunction, and hyperglycemia. Together, they drive the development of diabetic complications including nephropathy, retinopathy, and neurodegenerative disorders [8,9,10,11,12,13,14,15,16,17].

AGEs and the receptor for AGE (RAGE) play a central role in amplifying oxidative and inflammatory stress [8,18]. Activation of the AGE-RAGE pathway induces reactive oxygen and nitrogen species (ROS and RNS), partly via nicotinamide adenine dinucleotide phosphate (NADPH) oxidase and mitochondrial dysfunction [19].

This contributes to the formation of nitration and oxidation adducts such as 3-nitrotyrosine (3-NT), dityrosine (DT) and methionine sulfoxide (MetSO) [20]. Pro-inflammatory cascades further exacerbate tissue damage, notably through activation of nuclear factor kappa-light-chain-enhancer of activated B-cells (NF-κB) and transforming growth factor-β (TGFβ) signaling pathways [19]. These feedback loops promote sustained expression of pro-inflammatory cytokines (e.g., tumor necrosis factor (TNF)-α, interleukin (IL)-1β, IL-6) and RAGE overexpression, ultimately leading to persistent NF-κB activation and impaired resolution of inflammation [19,21,22,23,24,25,26].

AGEs are formed endogenously via the Maillard reaction or through highly reactive dicarbonyl intermediates such as methylglyoxal (MGO), glyoxal (GO), dimethylglyoxal (DMG), and 3-deoxyglucosone (3-DG), which originate from glucose autoxidation, lipid peroxidation, or fructose metabolism [8,27,28,29,30,31,32,33,34].

AGEs, nitration adducts, and oxidation adducts modify lysine, arginine, and tyrosine residues, impairing protein structure, enzymatic function, and posttranslational regulation [35,36,37,38]. Long-lived proteins in the extracellular matrix (ECM) of tissues like skin and bone are particularly susceptible to these reactive compounds due to their low turnover and limited antioxidant defenses [39]. Notable AGEs include methylglyoxal-derived hydroimidazolone (MG-H1), glyoxal-derived hydroimidazolone (G-H1), carboxyethyl-lysine (CEL), carboxymethyl-lysine (CML), fructosyl-lysine (FL) and glucosepane (GSP) [39,40,41,42,43].

In T2DM, elevated levels of these AGEs and their degradation products are observed in plasma and urine, where they may serve as biomarkers of metabolic stress and disease progression [43,44,45,46]. Free adducts are released during proteolysis and may be repaired, metabolized, or cleared renally [20,47].

Despite the clear contribution of the AGE-RAGE axis to diabetic complications, therapeutic strategies to interrupt this pathway remain underdeveloped [19].

Emerging evidence suggests that vitamin K2, particularly its subtypes menaquinone-4 (MK-4) and menaquinone-7 (MK-7), may exert beneficial effects on glucose metabolism by enhancing insulin secretion and modulating inflammatory responses through inhibition of NF-κB signaling [2,48,49,50,51,52,53,54,55].

However, MK-7 is superior to MK-4 with regard to its plasma half-life (approximately 70 h vs. ca. 2 h for MK-4) and its much higher intestinal absorption, markedly increasing its bioavailability [51,56,57]. Additionally, studies indicated that it exerts about 70% of its effects in extra-hepatic tissues or organs and shows a higher carboxylation co-factor activity than vitamin K1 and MK-4 [51,56,57]. Previous studies also determined that MK-4 supplementation did not raise MK-4 levels in serum or extrahepatic tissues and the authors concluded that MK-7 was the better choice for oral substitution and subsequent in vivo transformation to MK-4 than substituting MK-4 directly [58,59].

Given its antioxidant and anti-inflammatory properties, MK-7 has been proposed as a candidate for mitigating AGE-RAGE-mediated cellular stress [8]. However, whether MK-7 can modulate the accumulation of dicarbonyls, AGEs, and related nitration or oxidation adducts in diabetes mellitus remains largely unexplored.

To address this, we employed the Zucker Diabetic Fatty (ZDF) rat model, which harbors an autosomal-recessive leptin receptor gene mutation leading to obesity, insulin resistance, and overt T2DM in homozygous (fa/fa) males when fed a diabetogenic diet. Heterozygous animals (fa/+) remain metabolically healthy and serve as controls [60,61,62,63,64].

In this study, we investigated whether long-term dietary supplementation with MK-7 alters circulating and urinary levels of reactive dicarbonyls, free AGEs, and oxidative stress markers in hetero- and homozygous male ZDF rats.

## 2. Materials and Methods

### 2.1. Diabetic Animal Model, Sample Origin, and Ethical Considerations

Plasma and urine samples from twenty-four male ZDF rats (26- to 27-week-old, hetero- (fa/+, control) and homozygous (fa/fa, diabetic), Charles River Laboratories Inc., Châtillon-sur-Chalaronne, France) obtained in a prior study were used for this investigation. In brief, the animals were sourced 11 to 12 weeks of age and received a diabetogenic diet ad libitum. Starting at 14 to 15 weeks of age, half the animals in each group received 100 mg MK-7 (Kappa Bioscience AS, Oslo, Norway) per kg feed for the next 12 weeks until euthanization. The onset of T2DM was monitored using random testing with a veterinary glucometer (AlphaTrak 2, Zoetis, Tullytown, PA, USA) for whole blood samples.

During euthanization via left heart ventricle puncture with exsanguination in deep narcosis, blood, and urine samples (via bladder puncture) were collected into lithium-heparin and urine microtubes (Sarstedt, Nuembrecht, Germany).

The study adhered to Federation of European Laboratory Animal Science Associations (FELASA) and Animal Research: Reporting of In Vivo Experiments (ARRIVE) guidelines. Animal experimentation protocols were approved by the institutional review board and the regional animal review board (Regierung von Unterfranken: RUF 55.2.2-2532-2-729-17).

### 2.2. Sample Processing and Storage

After sampling, lithium-heparin, and urine microtubes were stored at 4 °C for a maximum of 4 to 8 h due to the batch-wise animal finalization. Lithium-heparin microtubes were centrifuged at 2000× *g* for 10 min, while urine microtubes were centrifuged at 400× *g* for 5 min. Supernatants were transferred to 1.5 or 2.0 mL Eppendorf tubes (Eppendorf Vertrieb Deutschland GmbH, Wesseling-Berzdorf, Germany) and frozen at −80 °C until use.

### 2.3. Measurement of Plasma Dicarbonyls

MGO, GO, 3-DG and DMG were determined by isotope dilution, tandem mass spectroscopy, following derivatization with 1,2-diaminobenzene (DB) [65,66].

Briefly, 20 µL plasma samples were acidified with ice-cold 20% (wt/vol) trichloroacetic acid (TCA; Sigma-Aldrich Chemie, Taufkirchen, Germany) in 10 µL 0.9% (wt/vol) sodium chloride (NaCl; Sigma-Aldrich Chemie), vortexed and diluted with 20 µL ultrapure water (Merck Chemicals GmbH, Darmstadt, Germany). A 5 µL aliquot of the internal standard (400 nM of [13C3]-MGO, [13C2]-GO, [13C6]-3-DG and [d6]-DMG) was then added and the samples vortexed again. Normal and isotopic standards were either purchased (Sigma-Aldrich Chemie and Santa Cruz Biotechnology, Dallas, TX, USA) or prepared in-house, as described previously [65]. Following centrifugation (20,000× *g* for 5 min at 4 °C), 35 µL of the supernatants was transferred to high performance liquid chromatography (HPLC) vials (VWR International, Radnor, PA, USA) containing a 200 µL glass insert (WICOM Germany GmbH, Heppenheim, Germany). A 5 µL aliquot of 3% sodium azide (wt/vol) (Sigma-Aldrich Chemie) was then added to each sample followed by 10 µL of 0.5 mM DB (Sigma-Aldrich Chemie) in 200 mM hydrochloric acid (HCl; Sigma-Aldrich Chemie) containing 0.5 mM diethylenetriaminepentaacetic acid (DETAPAC; Sigma-Aldrich Chemie) in liquid chromatography-mass spectrometry (LC-MS)-grade water (Carl Roth GmbH & Co. KG, Karlsruhe, Germany).

Samples were then incubated for 4 h at room temperature, protected from the light. Afterwards, they were analyzed by LC-MS/MS using an ACQUITY™ ultra-high-performance liquid chromatography system with a Xevo-TQS LC-MS/MS mass spectrometer (Waters Corporation, Manchester, UK). The column was a BEH C18 (1.7 µm particle size, 100 × 2.1 mm) and guard column (5 × 2.1 mm, Waters Corporation).

The mobile phase consisted of a mixture of LC-MS-grade water and acetonitrile (Carl Roth GmbH & Co. KG, Karlsruhe, Germany), with 0.1% formic acid (Biosolve B.V., Valkenswaard, The Netherlands) as a modifier: It started with 100% water containing 0.1% formic acid and transitioned to a mixture of 50% acetonitrile and 50% water containing 0.1% formic acid over 10 min using a linear gradient. The flow rate was 0.2 mL/min and column temperature was 5 °C. Dicarbonyls were detected by electrospray positive ionization with multiple reaction monitoring (MRM). The capillary voltage was 0.5 kV, the interscan delay time 100 ms, the source and desolvation gas temperatures 150 and 350 °C, respectively. Cone gas and desolvation gas flows were 150 L/h and 800 L/h. Molecular ion and fragment ion masses, as well as cone voltage and collision energy were optimized to ±0.1 Da and ±1 eV for MRM detection of the analytes (Supplementary Table S1). Acquisition and quantification were completed with MassLynx 4.1 and TargetLynx 2.7 (Waters Corporation).

### 2.4. Measurement of Plasma and Urine Free AGEs, Nitration and Oxidation Adducts

Plasma and urine free AGEs, nitration and oxidation adducts were determined by isotope dilution, tandem mass spectroscopy, as previously described [20].

Briefly, a 20 µL aliquot of plasma or urine was diluted to 500 µL with LC-MS-grade water (Carl Roth GmbH & Co. KG) and filtered by microspin ultrafiltration (10 kDa cut-off; Amicon, Merck Chemicals GmbH, Darmstadt, Germany) at 20,000× *g* for 30 min at 4 °C. The ultrafiltrate was then retained for the free adduct analysis. A 30 µL aliquot of the ultrafiltrate was spiked with an equal volume of 0.2% trifluoroacetic acid (TFA; Biosolve B.V., Valkenswaard, The Netherlands) in LC-MS-grade water (Carl Roth GmbH & Co. KG) containing the isotopic standards (1–50 pmol) and transferred to HPLC vials (VWR International) containing a 200 µL glass insert (WICOM Germany GmbH). Normal and isotopic standards were either purchased (Cambridge Isotope Laboratories, Tewksbury, MA, USA and Iris Biotech GmbH, Marktredwitz, Germany) or prepared in-house, as described previously [47,67].

Samples were then analyzed by LC-MS/MS using an ACQUITY ultra-high-performance liquid chromatography system with a Xevo-TQS LC-MS/MS spectrometer (Waters Corporation). Two 5 µm Hypercarb™ columns (Thermo Fisher Scientific, Waltham, MA, USA) in series were used: 2.1 × 50 mm, fitted with a 5 × 2.1 mm pre-column, and 2.1 × 250 mm. The mobile phases were 0.1% TFA (Biosolve B.V.) in water, and 0.1% TFA in 50% acetonitrile and 50% water. The column temperature and flow rate were 30 °C and 0.2 mL/min, respectively. Analytes were eluted using a two-step gradient and the columns washed after each sample with 0.1% TFA in 50% tetrahydrofuran (THF; Sigma-Aldrich Chemie, Taufkirchen, Germany), as described previously [20].

AGEs, oxidation and nitration markers were detected by electrospray positive ionization with MRM. The ionization source temperature was 150 °C and the desolvation temperature was 500 °C. The cone gas and desolvation gas flows were 150 L/h and 1000 L/h. The capillary voltage was 0.5 kV. Molecular ion and fragment ion masses, as well as cone voltage and collision energy were optimized to ±0.1 Da and ±1 eV for MRM detection of the analytes (Supplementary Table S1). Acquisition and quantification were completed with MassLynx 4.1 and TargetLynx 2 (Waters Corporation).

### 2.5. Statistical Testing

Statistical analyses were performed using GraphPad Prism 10.4.2 (GraphPad Software, San Diego, CA, USA). Data normality was assessed with the Shapiro–Wilk test, and outliers were detected using the “robust regression and outlier removal” (ROUT; Q = 1%) method. Results were presented as mean ± standard deviation (SD) in descriptive statistics tables. In graphs, they were presented as min-to-max plots with means indicated by the line in the middle of the plots. Group differences were analyzed using Welch’s analysis of variance (ANOVA) with Dunnett T3 multiple comparison test. Correlation analysis was performed using Pearson correlation coefficients (*r*) with two-tailed *t*-test for statistical significance. Confidence intervals (95% CI) were calculated for all correlations. Previously published data (body mass as well as MK-7, glucose and fructosamine serum concentrations) were included [68]. Correlation strength was categorized as “negligible” (*r* ≤ 0.10), “weak” (*r* = 0.11–0.39), “moderate” (*r* = 0.40–0.69), “strong” (*r* = 0.70–0.89) or “very strong (*r* ≥ 0.90), although these categorizations are not undisputed [69]. *p*-values < 0.05 were considered statistically significant.

## 3. Results

### 3.1. Plasma Dicarbonyls

**Figure 1:**
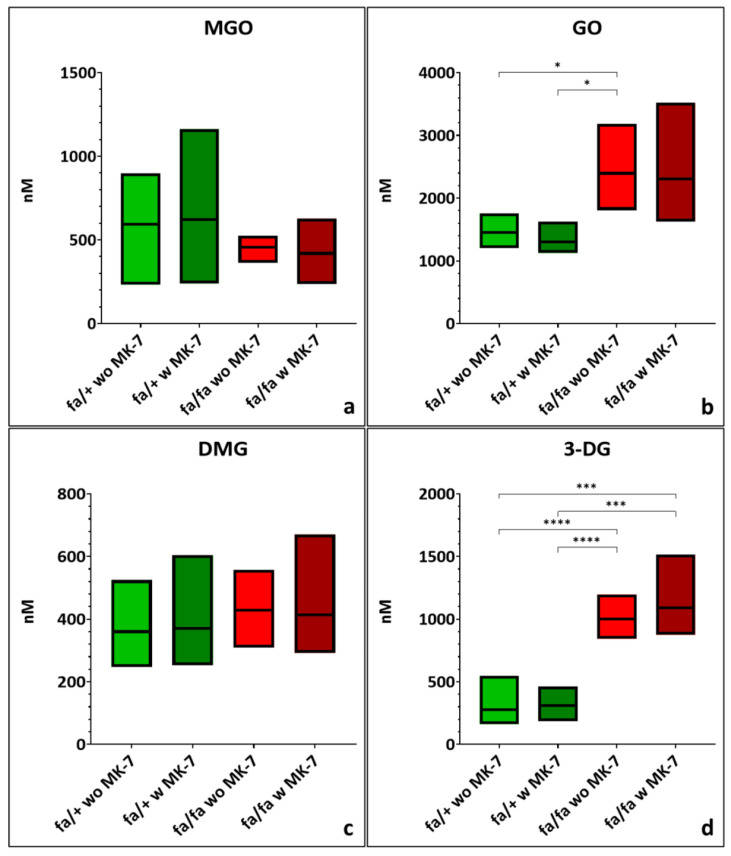
Quantification of methylglyoxal (MGO) (**a**), glyoxal (GO) (**b**), dimethylglyoxal (DMG) (**c**), and 3-deoxyglucosone (3-DG) (**d**) in lithium heparin plasma of hetero- and homozygous ZDF rats without or with menaquinone-7 (MK-7) supplementation; depicted as min-to-max plots, with means indicated by the line in the middle of the plots. *p*-values: * < 0.05, *** < 0.001, **** < 0.0001. fa/+: heterozygous ZDF rats; fa/fa: homozygous ZDF rats; w: with; wo: without. *n* = 5–6.

Mean plasma MGO concentrations were slightly lower in diabetic compared to non-diabetic rats, but the difference was not statistically significant (Figure 1a; Supplementary Table S2). GO was elevated in diabetic ZDF rats without MK-7 supplementation compared to non-diabetic rats (*p* < 0.05). Diabetic ZDF rats with MK-7 supplementation showed a similar GO increase but missed statistical significance (Figure 1b; Supplementary Table S2). DMG levels remained practically unchanged across all groups (Figure 1c; Supplementary Table S2). Plasma levels of 3-DG were elevated in both diabetic ZDF rat groups compared to non-diabetic controls (*p* < 0.001; Figure 1d; Supplementary Table S2). Diabetic rats displayed reduced variability (SD) in MGO levels and a greater variability in GO and 3-DG levels when compared to non-diabetic animals. MK-7 supplementation had no statistically significant effect on plasma levels of any of the four dicarbonyls in either genotype.

### 3.2. Plasma Free AGEs

**Figure 2:**
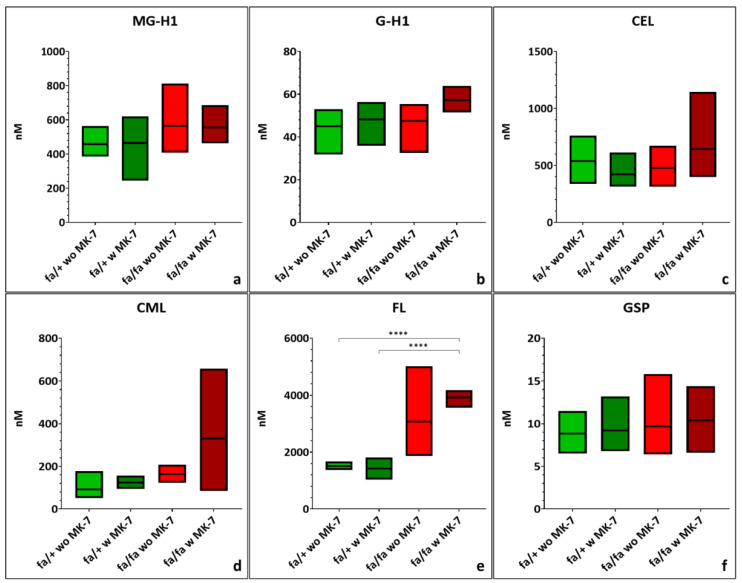
Quantification of free methylglyoxal-derived hydroimidazolone (MG-H1) (**a**), glyoxal-derived hydroimidazolone (G-H1) (**b**), carboxyethyl-lysine (CEL) (**c**), carboxymethyl-lysine (CML) (**d**), fructosyl-lysine (FL) (**e**) and glucosepane (GSP) (**f**) in lithium heparin plasma of hetero- and homozygous ZDF rats without or with menaquinone-7 (MK-7) supplementation depicted as min-to-max plots with means indicated by the line in the middle of the plots. *p*-values: **** < 0.0001. fa/+: heterozygous ZDF rats; fa/fa: homozygous ZDF rats, w: with, wo: without. *n* = 5–6.

Diabetic ZDF rats tended to have higher mean plasma MG-H1 levels compared to heterozygous controls, but this difference did not reach statistical significance and was unaffected by MK-7 supplementation (Figure 2a; Supplementary Table S2). MK-7 treatment in diabetic animals led to a non-significant increase in plasma G-H1, CEL, and CML levels, accompanied by greater interindividual variability in CEL and CML but reduced interindividual variability in G-H1 (Figure 2b–d; Supplementary Table S2). Plasma FL levels were higher in diabetic rats. MK-7 reduced variability in this diabetic group, leading to a statistically significant difference compared to both heterozygous subgroups (*p* < 0.0001; Figure 2e; Supplementary Table S2). GSP levels remained consistent across all groups and were largely unaffected by MK-7 (Figure 2f; Supplementary Table S2).

### 3.3. Plasma Free Nitration and Oxidation Adducts

**Figure 3:**
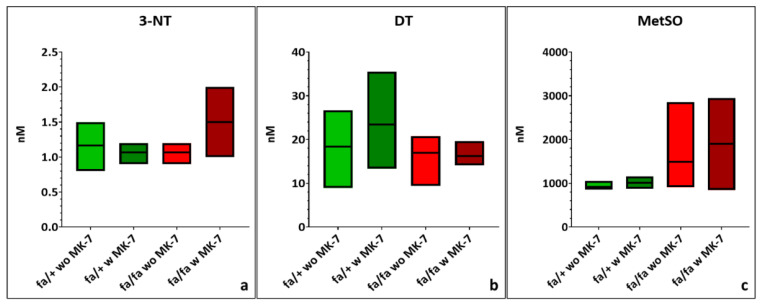
Quantification of 3-nitrotyrosine [3-NT] (**a**), dityrosine (DT) (**b**) and methionine-sulfoxide (MetSO) (**c**) in lithium heparin plasma of hetero- and homozygous ZDF rats without or with menaquinone-7 (MK-7) supplementation depicted as min-to-max plots with means indicated by the line in the middle of the plots. fa/+: heterozygous ZDF rats; fa/fa: homozygous ZDF rats, w: with, wo: without. *n* = 5–6.

Plasma concentrations of 3-NT were similar between diabetic and non-diabetic groups, but MK-7 supplementation increased 3-NT levels in diabetic rats. This effect was not statistically significant and was accompanied by increased variability (Figure 3a; Supplementary Table S2). DT levels were slightly lower in diabetic animals compared to controls, with a reduction in variability due to MK-7 in diabetic rats (Figure 3b; Supplementary Table S2). MetSO levels were elevated in diabetic rats, and MK-7 treatment led to a further, non-significant increase along with higher SD (Figure 3c; Supplementary Table S2).

### 3.4. Urinary Free AGEs

**Figure 4:**
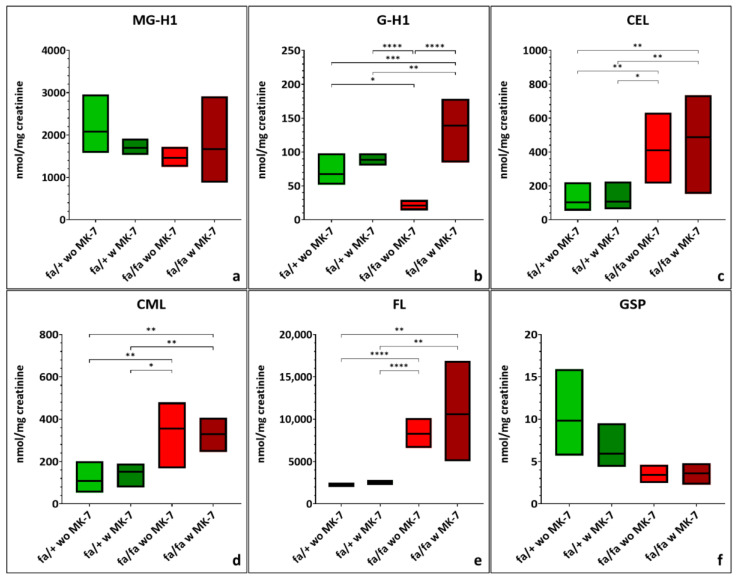
Quantification of free methylglyoxal-derived hydroimidazolone (MG-H1) (**a**), glyoxal-derived hydroimidazolone (G-H1) (**b**), carboxyethyl-lysine (CEL) (**c**), carboxymethyl-lysine (CML) (**d**), fructosyl-lysine (FL) (**e**) and glucosepane (GSP) (**f**) in urine of hetero- and homozygous ZDF rats without or with menaquinone-7 (MK-7) supplementation depicted as min-to-max plots with means indicated by the line in the middle of the plots. *p*-values: * < 0.05, ** < 0.01, *** < 0.001, **** < 0.0001. fa/+: heterozygous ZDF rats; fa/fa: homozygous ZDF rats, w: with, wo: without. *n* = 4–8.

Urinary MG-H1 levels did not differ significantly between diabetic and non-diabetic groups, regardless of MK-7 supplementation. Nonetheless, MK-7 supplementation increased variability in diabetic animals and reduced both the mean MG-H1 concentration and variability in non-diabetic controls (Figure 4a; Supplementary Table S3). In contrast, urinary G-H1 levels were lower in diabetic, non-supplemented rats but significantly elevated in MK-7-treated diabetic animals compared to both control groups (*p* < 0.05). MK-7 reduced variability in non-diabetic ZDF rats (Figure 4b; Supplementary Table S3). CEL concentrations were significantly higher in diabetic rats and further increased with MK-7 supplementation, associated with greater variability (*p* < 0.05; Figure 4c; Supplementary Table S3). Urinary CML levels were consistently elevated in diabetic rats, with reduced SD in the MK-7 group (*p* < 0.05; Figure 4d; Supplementary Table S3). FL concentrations were significantly increased in diabetic animals, associated with higher variability in the MK-7-supplemented rat group (*p* < 0.01; Figure 4e; Supplementary Table S3). GSP levels were lower in the urine of diabetic rats, with no effect of MK-7. However, MK-7 supplementation reduced both the mean GSP concentration and variability in heterozygous animals (Figure 4f; Supplementary Table S3).

### 3.5. Urinary Free Nitration and Oxidation Adducts

**Figure 5:**
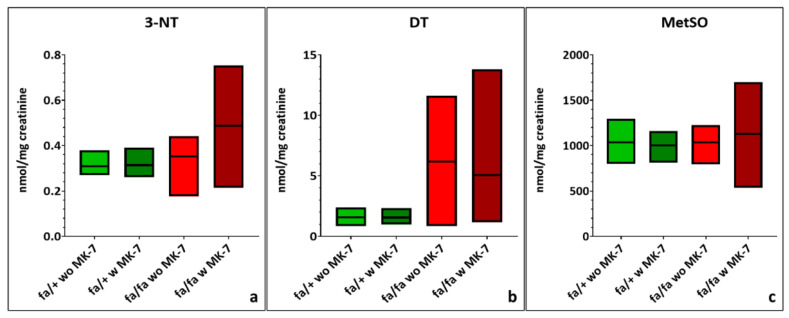
Quantification of 3-nitrotyrosine [3-NT] (**a**), dityrosine (DT) (**b**) and methionine-sulfoxide (MetSO) (**c**) in urine of hetero- and homozygous ZDF rats without or with menaquinone-7 (MK-7) supplementation depicted as min-to-max plots with means indicated by the line in the middle of the plots. fa/+: heterozygous ZDF rats; fa/fa: homozygous ZDF rats, w: with, wo: without. *n* = 3–8.

Urinary 3-NT levels were modestly increased (non-significant) in diabetic rats, with MK-7 supplementation resulting in a further non-significant increase (Figure 5a; Supplementary Table S3). DT concentrations were elevated (non-significant) and showed high variability in both diabetic groups (Figure 5b: Supplementary Table S3). Mean MetSO levels were similar across all groups (Figure 5c; Supplementary Table S3).

MK-7-treated diabetic rats consistently showed the highest variability for 3-NT, DT and MetSO concentrations.

### 3.6. Correlation Analysis

**Figure 6:**
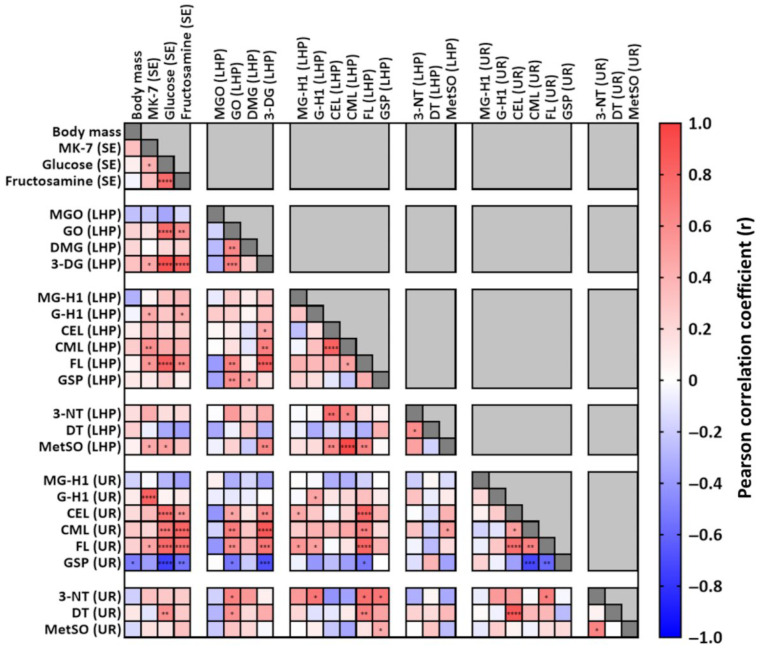
Correlation matrix of previously published data (body mass as well as menaquinone-7 [MK-7], glucose and fructosamine serum concentrations) [68], plasma levels of dicarbonyls (methylglyoxal [MGO], glyoxal [GO], dimethylglyoxal [DMG], and 3-deoxyglucosone [3-DG]), and plasma as well as urinary concentrations of advanced glycation end-products (methylglyoxal-derived hydroimidazolone [MG-H1], glyoxal-derived hydroimidazolone [G-H1], carboxyethyl-lysine [CEL], carboxymethyl-lysine [CML], fructosyl-lysine [FL], glucosepane [GSP]), and nitration (3-nitrotyrosine [3-NT]) and oxidation adducts (dityrosine [DT], methionine-sulfoxide [MetSO]). *p*-values: * < 0.05, ** < 0.01, *** < 0.001, **** < 0.0001. LHP: lithium heparin plasma, SE: serum, UR: urine.

Serum MK-7 concentrations exhibited moderate-to-strong positive correlations with serum glucose and multiple downstream markers of glycation and oxidative stress in both plasma and urine (Figure 6; Supplementary Table S4). The strongest associations were observed with plasma CML (*r* = 0.59, *p* < 0.01), FL (*r* = 0.55, *p* < 0.05), and urinary G-H1 (*r* = 0.82, *p* < 0.0001).

Serum glucose and fructosamine levels (Figure 6; Supplementary Table S4) were highly intercorrelated (*r* = 0.77, *p* < 0.0001) and showed consistent positive associations with plasma GO, 3-DG, and FL (glucose: *r* = 0.77–0.91, all *p* < 0.0001; fructosamine: *r* = 0.58–0.80, all *p* < 0.01), as well as with urinary CEL, CML, and FL (glucose: *r* = 0.71–0.79, all *p* < 0.001; fructosamine: *r* = 0.53–0.80, all *p* < 0.01).

Among the dicarbonyls, plasma GO and 3-DG (Figure 6; Supplementary Table S4) were strongly correlated with each other (*r* = 0.67, *p* < 0.001) and with multiple downstream AGEs. Both were positively associated with plasma FL (GO: *r* = 0.67, *p* < 0.01; 3-DG: *r* = 0.83, *p* < 0.0001), and with urinary CEL, CML, and FL (GO: *r* = 0.47–0.68, all *p* < 0.05; 3-DG: *r* = 0.62–0.87, all *p* < 0.01). Additional correlations for GO included plasma GSP (*r* = 0.59, *p* < 0.01), urinary 3-NT (*r* = 0.65, *p* < 0.05), and DT (*r* = 0.59–0.65, all *p* < 0.05). 3-DG was also associated with plasma CEL (*r* = 0.46, *p* < 0.05), CML (*r* = 0.67, *p* < 0.01), and MetSO (*r* = 0.62, *p* < 0.01).

Plasma MG-H1 and G-H1 were moderately associated with urinary FL (*r* = 0.50–0.55, all *p* < 0.05).

Plasma CEL and CML were strongly intercorrelated (*r* = 0.82, *p* < 0.0001) and both showed associations with oxidative markers, including plasma 3-NT (CEL: *r* = 0.76, *p* < 0.01; CML: *r* = 0.63, *p* < 0.05) and MetSO (CEL: *r* = 0.63, *p* < 0.01; CML: *r* = 0.94, *p* < 0.0001).

Plasma FL was positively correlated with plasma CML (*r* = 0.49, *p* < 0.05) and MetSO (*r* = 0.64, *p* < 0.01), and with urinary CEL, CML, 3-NT, and DT (*r* = 0.66–0.82, all *p* < 0.05).

Plasma GSP showed moderate associations with urinary 3-NT (*r* = 0.73, *p* < 0.05) and MetSO (*r* = 0.44, *p* < 0.05). Plasma 3-NT and DT were also moderately intercorrelated (*r* = 0.60, *p* < 0.05).

Finally, urinary GSP levels were inversely correlated with body mass, serum glucose, fructosamine, plasma GO, 3-DG, and FL (*r* = −0.75 to −0.52, all *p* < 0.05).

## 4. Discussion

In this study, we investigated the effects of high-dose MK-7 supplementation on plasma and urinary levels of dicarbonyls, AGEs, and protein nitration/oxidation adducts in diabetic and non-diabetic ZDF rats. Despite MK-7’s reported antioxidant and anti-inflammatory properties, we found no evidence of protective effects on any of the measured parameters. In some cases, particularly among AGEs and oxidation markers, MK-7 exacerbated inter-individual variability or elevated plasma and urinary levels in diabetic animals.

This contrasts with an in vitro mice/rat study as well as two in vivo studies in Wistar and Sprague-Dawley rats detailing beneficial effects of MK-4 on short-, mid- and long-term markers of glucose metabolism as well as insulin gene expression [70,71,72]. However, experimental setups in these studies differed markedly from our approach:

For the in vitro study, isolated pancreatic islets of mice and cells of a rat insulinoma cell line (INS-1) were stimulated with ≤20 µM MK-4 for a maximum of 1 h, leading to a statistically significant increase in insulin secretion in both cases [70]. The authors concluded that MK-4 might be a potent amplifier of incretins [70].

In the study of Hussein et al., male Wistar rats were included from 8 to 10 weeks of age and given a one-week acclimatization period. 4 weeks later, DMT2 was induced artificially via single intraperitoneal streptozotocin (STZ) injection. Seven days later, rats with ≥300 mg/dL plasma glucose were considered diabetic, Diabetic animals then received 10, 15, or 30 mg/kg bodyweight MK-4 via gavage daily over a period of 8 weeks until sacrifice [71]. The authors observed a dose-dependent decrease in fasting glucose while fasting insulin levels increased and hemoglobin A1c concentrations improved [71].

Seyama et al. used 7-week-old Sprague-Dawley rats and directly induced DMT2 via STZ [72]. Then they injected one subgroup of animals intraperitoneally with 100 mg/kg body weight vitamin K2 (subtype not specified) daily for 3 to 6 weeks in addition to an oral diet enriched with vitamin D2 [72]. After sacrifice, the authors determined that glucose and insulin plasma levels in the diabetic group receiving both vitamin D2 and vitamin K2 had largely returned to control levels and concluded that vitamin K2 improved glucose homeostasis [72]. The subgroup receiving estradiol instead of vitamin K2 showed largely the same results regarding plasma glucose and insulin levels, which was attributed to the radical scavenging activity of both estradiol and vitamin K2 [72].

In our study, MK.7 supplementation of male ZDF rats via ad libitum feeding started at 14 to 15 weeks of age and lasted for 12 weeks. Additionally, we used a genetic model of DMT2 instead of STZ-induced DMT2.

The different outcomes between our study and the cited in vivo studies could be grounded in these methodological differences. Additionally, results of in vitro experiments are not necessarily transferable to in vivo situations and gene expression and post-transcriptional processing of insulin differ between mice and rats [73].

Dicarbonyl compounds such MGO, GO, and 3-DG are critical precursors in AGE formation and known contributors to diabetic complications, including inflammation via increased expression of pro-inflammatory cytokines such as TNF-α and IL-1, endothelial dysfunction, nephropathy, and neuropathy [66,74,75,76,77,78,79]. In our model, diabetic rats displayed significantly higher plasma GO and 3-DG levels, with a trend toward reduced MGO. These findings align with known differences in glyoxalase-mediated detoxification: MGO is rapidly cleared via the glyoxalase 1/glyoxalase 2 (GLO1/GLO2) system, whereas GO and 3-DG rely more heavily on alternative, less efficient detoxification pathways [80,81,82,83,84,85,86]. Additionally, DMG is a highly reactive and neurotoxic dicarbonyl with localized generation as well as a very short half-life [66].

The MGO, GO and 3-DG concentrations detected in our study are higher than the reported normal plasma levels in non-diabetic humans, possibly due to hemolysis in a subset of samples [43,65]. Inter-species differences might also have contributed to this observation.

Downstream AGEs reflected similar patterns to dicarbonyls: Plasma levels of MG-H1, CEL, CML, and FL were elevated in diabetic animals, consistent with previous reports despite substantial interindividual variability [40,47,87]. Urinary excretion of several AGEs (CEL, CML, FL) was also increased, suggesting enhanced renal clearance, possibly as a compensatory mechanism in response to elevated systemic glycation burden [83,88,89,90].

Correlation analyses indicated that elevated plasma AGE levels were primarily driven by the increased formation of reactive dicarbonyl precursors under hyperglycemic conditions, rather than by impaired detoxification. Specifically, serum glucose and fructosamine levels showed strong positive associations with plasma GO and 3-DG—precursors of CEL, CML, and FL—which, in turn, were significantly correlated with their respective AGEs in both plasma and urine [76,77,78,91,92,93]. Positive associations between AGEs and markers of oxidative stress further suggest convergence of glycation and redox pathways.

Urinary GSP levels were inversely correlated with serum glucose, fructosamine, and several dicarbonyl and AGE species (GO, 3-DG, FL), suggesting that clearance pathways may be overwhelmed or downregulated under sustained glycation stress. While this finding points to compromised detoxification capacity, it likely reflects a secondary effect rather than a primary driver of AGE accumulation.

MK-7 supplementation did not attenuate any of these metabolic changes in our animal model. On the contrary, diabetic rats receiving MK-7 exhibited non-significant increases in several AGEs (G-H1, CEL, CML), and a significant elevation of FL in both plasma and urine. These trends, coupled with increased variability, reflect either enhanced oxidative stress not mitigated by MK-7, altered metabolic fluxes promoting AGE formation or even direct oxidative damage via MK-7 since the redox cycling properties of menadione (vitamin K3) might deplete glutathione (GSH), especially in individuals of advanced age, leading to decreased activity of GSH-dependent detoxification enzymes [94]. Since all vitamin K forms are converted to MK-4 via menadione and MK-7 serum concentrations were very high in supplemented homozygous ZDF rats, the active increase in oxidative damage via MK-7 appears as a relevant possibility even though we did not detect any signs of overt toxicity in our previous study and other authors found no toxicity after application of up to 4500 mg/kg bodyweight over a period of 90 days or single dose of 5000 mg/kg body weight in Sprague-Dawley rats [68,95,96,97]. A certain degree of GSH depletion is supported by our observations of increased serum and urine levels of 3-NT and MetSO in MK-7-supplemented diabetic ZDF rats since GSH plays a preventive role in 3-NT formation and a supporting role in reductase systems limiting MetSO formation [98,99].

However, while MGO plasma levels in MK-7-supplemented diabetic ZDF rats showed an increased variability, the mean value was not higher than in non-supplemented homozygous animals. Since MGO is only efficiently detoxified by the glyoxalase (GLO) system, sufficient GSH concentrations for the continued activity of GLO-1 and GLO-2 were apparently still available [83].

Nonetheless, considering our observations and the available scientific data by other authors, potential adverse consequences of (high-dose) MK-7 supplementation including a possible increase in oxidative stress, especially in elderly experimental animals and humans, should be contemplated for future studies: Given that elevated circulating levels of G-H1, CEL, CML and FL have been linked to arterial stiffening, cardiovascular disease, and bone fragility in individuals with diabetes, high-dose MK-7 supplementation may aggravate glycation-associated complications like inflammation, endothelial dysfunction, and atherosclerotic changes in this context [34,39,42,45,100,101,102,103,104].

In contrast to other AGEs, urinary G-H1 levels were reduced in diabetic, non-supplemented ZDF rats. This could reflect differential renal handling, reduced excretion, or increased tissue retention of G-H1 in the diabetic state. Further studies will be needed to clarify the mechanisms underlying this distinct pattern.

While our study provides valuable insight into circulating and excreted markers, it may underestimate MK-7’s potential effects on tissue-resident AGEs and ECM remodeling. Circulating levels of dicarbonyls and AGEs are influenced not only by production and clearance but also by protein turnover and tissue remodeling. Long-lived proteins such as collagen are especially vulnerable to glycation and oxidation and important in the development of cardiovascular disease, nephropathy, and osteoporosis in patients with T2DM. However, their degradation products may not appear promptly in plasma or urine [8,39,40,44,105,106,107]. In this context, the stable plasma levels of glucosepane, a collagen crosslinking AGE, likely reflect the slow turnover of ECM components rather than true absence of change [41,108].

Further investigation into tissue-specific AGE deposition (e.g., in liver, kidney, and pancreas) and the regulatory role of AGE receptors (RAGE, sRAGE, AGER1) are necessary to fully elucidate the scope of MK-7’s metabolic impact. This could include experiments with altered supplementation durations, MK-7 concentrations or possibly combination therapies.

Additionally, as discussed in our previous article, (immuno-)histological assessments as well as investigations into signaling pathways like small mothers against decapentaplegic homolog (SMAD)-2 and -3, matrix-Gla protein (MGP), osteocalcin (OC), cytokine signaling will be needed to further supplement our understanding of overall MK-7 effects in T2DM [68].

## 5. Conclusions

In this model of DMT2, MK-7 supplementation did not attenuate circulating or urinary levels of dicarbonyls, AGEs, or nitration/oxidation adducts. On the contrary, several markers showed increased variability or upward trends in MK-7-treated diabetic rats. These findings suggest that MK-7, at the dose and duration tested, does not significantly ameliorate dicarbonyl or glycoxidative stress in ZDF rats. In prospect of available scientific evidence, our observations even point to a certain degree of aggravation of the metabolic dysfunction. However, our findings do not exclude tissue-specific or receptor-mediated effects not captured by the current experimental design. Additionally, the high inter-individual variability observed—particularly among MK-7-supplemented diabetic rats—raises the possibility that MK-7’s effects are more nuanced or context-dependent than initially assumed. Whether this variability reflects differences in disease progression or redox state requires further investigation.

Our results highlight the complexity of antioxidant strategies in diabetes and the importance of carefully selecting endpoints that align with the proposed mechanism of action. While MK-7 remains a compound of interest for metabolic and vascular health, further research is needed to clarify its precise targets and efficacy in settings of advanced glycation and oxidative damage.

To our knowledge, this is the first study to simultaneously assess plasma and urine levels of a wide panel of dicarbonyls, free AGEs, and nitration/oxidation adducts in the ZDF rat model. This comprehensive approach enables insight into both systemic metabolic stress and renal clearance patterns under diabetic conditions.

The above-mentioned high-physiological inter-individual variability and the presence of hemolysis in some samples, which may have influenced plasma dicarbonyl values, represent limitations of our study. Additionally, dose-dependent MK-7 effects, as well as tissue-level changes, were not investigated and the relatively small sample size limited statistical power to detect more subtle effects.

## Data Availability

The data presented in this study are openly available in Mendeley Data at https://doi.org/10.17632/vymyhk42th.1.

## Supplementary Materials

The following supporting information can be downloaded at: https://www.mdpi.com/article/10.3390/nu17172733/s1, Supplementary Table S1: Mass Transitions used for Multiple-Reaction Monitoring (MRM) of methylglyoxal (MGO), glyoxal (GO), dimethylglyoxal (DMG), 3-deoxyglucosone (3-DG), methylglyoxal-derived hydroimidazolone (MG-H1), glyoxal-derived hydroimidazolone (G-H1), carboxyethyl-lysine (CEL), carboxymethyl-lysine (CML), fructosyl-lysine (FL), glucosepane (GSP), 3-nitrotyrosine (3-NT), dityrosine (DT), and methionine sulfoxide (MetSO).; Supplementary Table S2: Descriptive statistics of plasma free levels of methylglyoxal (MGO), glyoxal (GO), dimethylglyoxal (DMG), 3-deoxyglucosone (3-DG), methylglyoxal-derived hydroimidazolone (MG-H1), glyoxal-derived hydroimidazolone (G-H1), carboxyethyl-lysine (CEL), carboxymethyl-lysine (CML), fructosyl-lysine (FL), glucosepane (GSP), 3-nitrotyrosine (3-NT), dityrosine (DT) and methionine sulfoxide (MetSO) in hetero- and homozygous ZDF rats without or with menaquinone-7 (MK-7) supplementation.; Supplementary Table S3: Descriptive statistics of urine levels of methylglyoxal-derived hydroimidazolone (MG-H1), glyoxal-derived hydroimidazolone (G-H1), carboxyethyl-lysine (CEL), carboxymethyl-lysine (CML), fructosyl-lysine (FL), glucosepane (GSP), 3-nitrotyrosine (3-NT), dityrosine (DT) and methionine-sulfoxide (MetSO) in hetero- and homozygous ZDF rats without or with menaquinone-7 (MK-7) supplementation.; Supplementary Table S4: Pearson correlation coefficients (*r*), their 95% confidence intervals (CI) and strength of statistically significant correlations of body mass as well as menaquinone-7 (MK-7), glucose and fructosamine serum concentrations, plasma levels of methylglyoxal (MGO), glyoxal (GO), dimethylglyoxal (DMG) and 3-deoxyglucosone (3-DG) as well as plasma and urinary concentrations of methylglyoxal-derived hydroimidazolone (MG-H1), glyoxal-derived hydroimidazolone (G-H1), carboxyethyl-lysine (CEL), carboxymethyl-lysine (CML), fructosyl-lysine (FL), glucosepane (GSP), 3-nitrotyrosine (3-NT), dityrosine (DT) and methionine-sulfoxide (MetSO) in hetero- and homozygous ZDF rats without or with menaquinone-7 (MK-7) supplementation.

## Abbreviations

The following abbreviations are used in this manuscript:

3-DG	3-deoxyglucosone
3-NT	3-nitrotyrosine
AGE(s)	Advanced glycation end-product(s)
ANOVA	Analysis of variance
ARRIVE	Animal Research: Reporting of In Vivo Experiments
CEL	Carboxyethyl-lysine
CI	Confidence interval
CML	Carboxymethyl-lysine
DB	1,2-diaminobenzene
DETAPAC	Diethylenetriaminepentaacetic acid
DMG	Dimethylglyoxal
DT	Dityrosine
ECM	Extracellular matrix
FELASA	Federation of European Laboratory Animal Science Associations
FL	Fructosyl-lysine
G-H1	Glyoxal-derived hydroimidazolone
GO	Glyoxal
GSP	Glucosepane
HCl	Hydrochloric acid
HPLC	High performance liquid chromatography
IL	Interleukin
IL-1β	Interleukin-1β
IL-6	Interleukin-6
LC-MS	Liquid chromatography-mass spectrometry
LHP	Lithium heparin plasma
MG-H1	Methylglyoxal-derived hydroimidazolone
MGO	Methylglyoxal
MK-4	Menaquinone-4
MK-7	Menaquinone-7
MRM	Multiple reaction monitoring
NaCl	Sodium chloride
NADPH	Nicotinamide adenine dinucleotide phosphate
NF-κB	Nuclear factor kappa-light-chain-enhancer of activated B-cells
RAGE	Receptor for AGE
RNS	Reactive nitrogen species
ROS	Reactive oxygen species
ROUT	Robust regression and outlier removal
RUF	Regierung von Unterfranken
SD	Standard deviation
SE	Serum
STZ	Streptozotocin
TCA	Trichloroacetic acid
TFA	Trifluoroacetic acid
THF	Tetrahydrofuran
TNF-α	Tumor necrosis factor-α
UR	Urine
ZDF	Zucker diabetic fatty
