# Supplementary material for: Menaquinone-7 Supplementation Increases Multiple Advanced Glycation End-Products and Oxidation Markers in Zucker Diabetic Fatty Rats
Source: Nutrients. 2025 Aug 23;17(17):2733. doi: 10.3390/nu17172733 (PMC12430629; doi:10.3390/nu17172733)
Supplement: Supplementary file 1 [file nutrients-17-02733-s001.zip › Supplementary Table S1 - 21.08.2025.pdf]

Supp. Tab. S1: Mass Transitions used for Multiple-Reaction Monitoring (MRM) of methylglyoxal (MGO), glyoxal (GO), dimethylglyoxal (DMG), 3-deoxyglucosone (3-DG), methylglyoxal-derived hydroimidazolone (MG-H1), glyoxal-derived hydroimidazolone (G-H1), carboxyethyl-lysine (CEL), carboxymethyl-lysine (CML), fructosyl-lysine (FL), glucosepane (GSP), 3-nitrotyrosine (3-NT), dityrosine (DT), and methionine sulfoxide (MetSO).

Analyte	Molecular Ion (m/z)	Fragment Ion (m/z)	Cone Voltage (V)	Collision Energy (eV)
MGO	145.0	76.9	10.0	24.0
GO	130.9	77.0	10.0	22.0
DMG	159.1	76.8	18.0	28.0
3-DG	235.1	199.1	10.0	16.0
MG-H1	229.1	113.4	20.0	14.0
G-H1	215.2	102.1	29.0	16.0
CEL	219.1	130.1	21.0	15.0
CML	205.0	130.0	24.0	15.0
FL	291.2	84.2	43.0	30.0
GSP	429.3	269.1	42.0	35.0
3-NT	227.1	180.9	20.0	20.0
DT	361.1	315.2	32.0	20.0
MetSO	166.0	73.9	16.0	14.0
