# Supplementary material for: Menaquinone-7 Supplementation Increases Multiple Advanced Glycation End-Products and Oxidation Markers in Zucker Diabetic Fatty Rats
Source: Nutrients. 2025 Aug 23;17(17):2733. doi: 10.3390/nu17172733 (PMC12430629; doi:10.3390/nu17172733)
Supplement: Supplementary file 1 [file nutrients-17-02733-s001.zip › Supplementary Table S2 - 21.08.2025.pdf]

Supp. Tab. S2: Descriptive statistics of plasma free levels of methylglyoxal (MGO), glyoxal (GO), dimethylglyoxal (DMG), 3-deoxyglucosone (3-DG), methylglyoxal-derived hydroimidazolone (MG-H1), glyoxal-derived hydroimidazolone (G-H1), carboxyethyl-lysine (CEL), carboxymethyl-lysine (CML), fructosyl-lysine (FL), glucosepane (GSP), 3-nitrotyrosine (3-NT), dityrosine (DT) and methionine sulfoxide (MetSO) in hetero- and homozygous ZDF rats without or with menaquinone-7 (MK-7) supplementation.

	**fa/+ wo MK-7**	**fa/+ w MK-7**	**fa/fa wo MK-7**	**fa/fa w MK-7**	
**n (outliers)**	6	6	6	6	
**mean**	593	621	455	147	**MGO (nM)**
**standard deviation**	123	158	26	60	
**n (outliers)**	6	6 (1)	6	6 (1)	
**mean**	1,453	1,300	2,396	2,309	**GO (nM)**
**standard deviation**	220	217	537	777	
**n (outliers)**	6	6	6	6	
**mean**	360	371	429	414	**DMG (nM)**
**standard deviation**	103	127	110	137	
**n (outliers)**	6	6	6	6	
**mean**	278	310	999	1,090	**3-DG (nM)**
**standard deviation**	141	108	115	240	
**n (outliers)**	6	6	6	6	
**mean**	458	465	565	556	**MG-H1 (nM)**
**standard deviation**	74.9	142	134	74.2	
**n (outliers)**	6	6	6	6 (1)	
**mean**	45.0	48.3	47.4	57.1	**G-H1 (nM)**
**standard deviation**	7.8	7.2	7.9	4.5	
**n (outliers)**	6	6	6	6	**CEL (nM)**
**mean**	536	422	477	644	
**standard deviation**	164	100	125	278	
**n (outliers)**	6 (1)	6 (1)	6 (1)	6	**CML (nM)**
**mean**	91.1	124	163	330	
**standard deviation**	51.6	23.9	35.3	260	
**n (outliers)**	6 (1)	6 (1)	G (1)	6 (1)	
**mean**	1,502	1,428	3,075	3,913	**FL (nM)**
**standard deviation**	118	343	1,198	250	
**n (outliers)**	6	6	6	6	
**mean**	8.9	9.2	9.7	10.4	**GSP (nM)**
**standard deviation**	1.8	2.5	3.7	3.0	
**n (outliers)**	3	3	3	3	
**mean**	1.17	1.07	1.07	1.5	**3-NT (nM)**
**standard deviation**	0.35	0.15	0.15	0.5	
**n (outliers)**	6	6	6	6	
**mean**	18.4	23.4	17.0	16.2	**DT (nM)**
**standard deviation**	6.4	8.1	4.2	2.4	
**n (outliers)**	6 (1)	6 (1)	6	6	
**mean**	919	1,010	1,488	1,902	**MetSO (nM)**
**standard deviation**	77.6	127	720	1,065	

fa/+: heterozygous ZDF rats; fa/fa: homozygous ZDF rats, w: with, wo: without.
