# Supplementary material for: Menaquinone-7 Supplementation Increases Multiple Advanced Glycation End-Products and Oxidation Markers in Zucker Diabetic Fatty Rats
Source: Nutrients. 2025 Aug 23;17(17):2733. doi: 10.3390/nu17172733 (PMC12430629; doi:10.3390/nu17172733)
Supplement: Supplementary file 1 [file nutrients-17-02733-s001.zip › Supplementary Table S3 - 21.08.2025.pdf]

Supp. Tab. S3: Descriptive statistics of urine levels of methylglyoxal-derived hydroimidazolone (MG-H1), glyoxal-derived hydroimidazolone (G-H1), carboxyethyl-lysine (CEL), carboxymethyl-lysine (CML), fructosyl-lysine (FL), glucosepane (GSP), 3-nitrotyrosine (3-NT), dityrosine (DT) and methionine-sulfoxide (MetSO) in hetero- and homozygous ZDF rats without or with menaquinone-7 (MK-7) supplementation.

	**fa/+ wo MK-7**	**fa/+ w MK-7**	**fa/fa wo MK-7**	**fa/fa w MK-7**	
**n (outliers)**	5	4	7	8	**MG-H1**
**mean**	2,077	1,699	1,460	1,672	**(nmol/mg**
**standard deviation**	535	169	183	657	**creatinine)**
**n (outliers)**	5	4	7	8	**G-H1**
**mean**	67.3	88.7	20.9	139	**(nmol/mg**
**standard deviation**	18.4	7.39	4.94	27.8	**creatinine)**
**n (outliers)**	5	4	7	8	**CEL**
**mean**	101	106	410	487	**(nmol/mg**
**standard deviation**	71.6	79.9	165	204	**creatinine)**
**n (outliers)**	5	4	7	6 (2)	**CML**
**mean**	109	152	355	329	**(nmol/mg**
**standard deviation**	59.3	52.2	118	62.5	**creatinine)**
**n (outliers)**	5	4	7	8	**FL**
**mean**	2,149	2,498	8,286	10,550	**(nmol/mg**
**standard deviation**	209	281	1,206	3,558	**creatinine)**
**n (outliers)**	5	4	6 (1)	8	**GSP**
**mean**	9.82	5.93	3.44	3.60	**(nmol/mg**
**standard deviation**	4.33	2.41	0.76	0.98	**creatinine)**
**n (outliers)**	3	3	3	3	**3-NT**
**mean**	0.31	0.31	0.35	0.49	**(nmol/mg**
**standard deviation**	0.06	0.07	0.15	0.27	**creatinine)**
**n (outliers)**	4 (1)	4	7	5 (3)	**DT**
**mean**	1.57	1.55	6.18	5.04	**(nmol/mg**
**standard deviation**	0.75	0.67	4.25	5.24	**creatinine)**
**n (outliers)**	5	4	7	8	**MetSO**
**mean**	1,036	1,002	1,036	1,128	**(nmol/mg**
**standard deviation**	215	168	145	389	**creatinine)**

fa/+: heterozygous ZDF rats; fa/fa: homozygous ZDF rats, w: with, wo: without.
