# Supplementary material for: Menaquinone-7 Supplementation Increases Multiple Advanced Glycation End-Products and Oxidation Markers in Zucker Diabetic Fatty Rats
Source: Nutrients. 2025 Aug 23;17(17):2733. doi: 10.3390/nu17172733 (PMC12430629; doi:10.3390/nu17172733)
Supplement: Supplementary file 1 [file nutrients-17-02733-s001.zip › Supplementary Table S4 - 21.08.2025.pdf]

Supp. Tab. S4: Pearson correlation coefficients (*r*), their 95% confidence intervals (CI) and strength of statistically significant correlations of body mass as well as menaquinone-7 (MK-7), glucose and fructosamine serum concentrations, plasma levels of methylglyoxal (MGO), glyoxal (GO), dimethylglyoxal (DMG) and 3-deoxyglucosone (3-DG) as well as plasma and urinary concentrations of methylglyoxal-derived hydroimidazolone (MG-H1), glyoxal-derived hydroimidazolone (G-H1), carboxyethyl-lysine (CEL), carboxymethyl-lysine (CML), fructosyl-lysine (FL), glucosepane (GSP), 3-nitrotyrosine (3-NT), dityrosine (DT) and methionine-sulfoxide (MetSO) in hetero- and homozygous ZDF rats without or with menaquinone-7 (MK-7) supplementation.

	r	95% CI	*p*-value	Strength
**Body mass & GSP (UR)**	-0.52	-0.77 – -0.11	0.017	moderate
**MK-7 (SE) & glucose (SE)**	0.42	0.06 – 0.68	0.025	moderate
**MK-7 (SE) & 3-DG (LHP)**	0.46	0.07 – 0.73	0.025	moderate
**MK-7 (SE) & G-H1 (LHP)**	0.45	0.04 – 0.73	0.033	moderate
**MK-7 (SE) & CML (LHP)**	0.59	0.21 – 0.81	0.005	moderate
**MK-7 (SE) & FL (LHP)**	0.55	0.14 – 0.80	0.013	moderate
**MK-7 (SE) & MetSO (LHP)**	0.45	0.03 – 0.73	0.038	moderate
**MK-7 (SE) & G-H1 (UR)**	0.82	0.62 – 0.92	< 0.0001	strong
**MK-7 (SE) & FL (UR)**	0.48	0.09 – 0.74	0.019	moderate
**Glucose (SE) & fructosamine (SE)**	0.77	0.57 – 0.89	< 0.0001	strong
**Glucose (SE) & GO (LHP)**	0.77	0.51 – 0.90	< 0.0001	strong
**Glucose (SE) & 3-DG (LHP)**	0.91	0.79 – 0.96	< 0.0001	very strong
**Glucose (SE) & FL (LHP)**	0.85	0.64 – 0.94	< 0.0001	strong
**Glucose (SE) & MetSO (LHP)**	0.50	0.08 – 0.77	0.022	moderate
**Glucose (SE) & CEL (UR)**	0.77	0.52 – 0.90	< 0.0001	strong
**Glucose (SE) & CML (UR)**	0.71	0.40 – 0.87	< 0.001	strong
**Glucose (SE) & FL (UR)**	0.79	0.55 – 0.91	< 0.0001	strong
**Glucose (SE) & GSP (UR)**	-0.75	-0.89 – -0.48	< 0.0001	strong
**Glucose (SE) & DT (UR)**	0.59	0.19 – 0.82	0.008	moderate
**Fructosamine (SE) & GO (LHP)**	0.58	0.20 – 0.81	0.006	moderate
**Fructosamine (SE) & 3-DG (LHP)**	0.80	0.58 – 0.91	< 0.0001	strong
**Fructosamine (SE) & G-H1 (LHP)**	0.43	0.01 – 0.72	0.044	moderate
**Fructosamine (SE) & FL (LHP)**	0.62	0.23 – 0.84	0.005	moderate
**Fructosamine (SE) & CEL (UR)**	0.53	0.16 – 0.78	0.009	moderate
**Fructosamine (SE) & CML (UR)**	0.80	0.57 – 0.92	< 0.0001	strong
**Fructosamine (SE) & FL (UR)**	0.73	0.46 – 0.88	< 0.0001	strong
**Fructosamine (SE) & GSP (UR)**	-0.56	-0.79 – -0.18	0.007	moderate
**GO (LHP) & DMG (LHP)**	0.62	0.27 – 0.83	0.002	moderate
**GO (LHP) & 3-DG (LHP)**	0.67	0.35 – 0.85	0.001	moderate
**GO (LHP) & FL (LHP)**	0.67	0.30 – 0.87	0.002	moderate
**GO (LHP) & GSP (LHP)**	0.59	0.22 – 0.81	0.004	moderate
**GO (LHP) & CEL (UR)**	0.47	0.02 – 0.76	0.042	moderate
**GO (LHP) & CML (UR)**	0.68	0.31 – 0.87	0.002	moderate
**GO (LHP) & FL (UR)**	0.60	0.21 – 0.83	0.006	moderate
**GO (LHP) & GSP (UR)**	-0.54	-0.80 – -0.10	0.021	moderate
**GO (LHP) & 3-NT (UR)**	0.65	0.04 – 0.91	0.042	moderate
**GO (LHP) & DT (UR)**	0.59	0.13 – 0.84	0.017	moderate

**DMG (LHP) & GSP (LHP)**	0.44	0.04 – 0.71	0.033	moderate
**3-DG (LHP) & CEL (LHP)**	0.46	0.07 – 0.73	0.023	moderate
**3-DG (LHP) & CML (LHP)**	0.67	0.33 – 0.85	0.001	moderate
**3-DG (LHP) & FL (LHP)**	0.83	0.61 – 0.93	< 0.0001	strong
**3-DG (LHP) & MetSO (LHP)**	0.62	0.27 – 0.83	0.002	moderate
**3-DG (LHP) & CEL (UR)**	0.62	0.26 – 0.83	0.003	moderate
**3-DG (LHP) & CML (UR)**	0.87	0.70 – 0.95	< 0.0001	strong
**3-DG (LHP) & FL (UR)**	0.73	0.41 – 0.88	< 0.001	strong
**3-DG (LHP) & GSP (UR)**	-0.69	-0.87 – -0.36	< 0.001	moderate
**MG-H1 (LHP) & CEL (UR)**	0.45	0.02 – 0.74	0.041	moderate
**MG-H1 (LHP) & FL (UR)**	0.55	0.15 – 0.79	0.010	moderate
**G-H1 (LHP) & G-H1 (UR)**	0.47	0.04 – 0.76	0.036	moderate
**G-H1 (LHP) & FL (UR)**	0.50	0.07 – 0.77	0.025	moderate
**G-H1 (LHP) & 3-NT (UR)**	0.72	0.17 – 0.93	0.019	strong
**CEL (LHP) & CML (LHP)**	0.82	0.60 – 0.92	< 0.0001	strong
**CEL (LHP) & 3-NT (LHP)**	0.76	0.34 – 0.93	0.004	strong
**CEL (LHP) & MetSO (LHP)**	0.63	0.28 – 0.83	0.002	moderate
**CML (LHP) & FL (LHP)**	0.49	0.04 – 0.77	0.034	moderate
**CML (LHP) & 3-NT (LHP)**	0.63	0.10 – 0.89	0.027	moderate
**CML (LHP) & MetSO (LHP)**	0.94	0.86 – 0.98	< 0.0001	very strong
**FL (LHP) & MetSO (LHP)**	0.64	0.27 – 0.84	0.003	moderate
**FL (LHP) & CEL (UR)**	0.82	0.57 – 0.93	< 0.0001	strong
**FL (LHP) & CML (UR)**	0.68	0.29 – 0.87	0.003	moderate
**FL (LHP) & FL (UR)**	0.80	0.54 – 0.92	< 0.0001	strong
**FL (LHP) & GSP (UR)**	-0.54	-0.80 – -0.09	0.022	moderate
**FL (LHP) & 3-NT (UR)**	0.75	0.17 – 0.94	0.020	strong
**FL (LHP) & DT (UR)**	0.66	0.21 – 0.88	0.010	moderate
**GSP (LHP) & 3-NT (UR)**	0.73	0.20 – 0.92	0.013	strong
**GSP (LHP) & MetSO (UR)**	0.44	0.01 – 0.73	0.044	moderate
**3-NT (LHP) & DT (LHP)**	0.60	0.03 – 0.87	0.041	moderate
**MetSO (LHP) & CML (UR)**	0.52	0.08 – 0.79	0.024	moderate
**CEL (UR) & CML (UR)**	0.51	0.11 – 0.77	0.016	moderate
**CEL (UR) & FL (UR)**	0.75	0.50 – 0.89	< 0.0001	strong
**CEL (UR) & DT (UR)**	0.87	0.70 – 0.95	< 0.0001	strong
**CML (UR) & FL (UR)**	0.65	0.32 – 0.84	0.001	moderate
**CML (UR) & GSP (UR)**	-0.72	-0.88 – -0.42	< 0.001	strong
**FL (UR) & GSP (UR)**	-0.60	-0.81 – -0.25	0.003	moderate
**FL (UR) & 3-NT (UR)**	0.68	0.17 – 0.90	0.016	moderate
**3-NT (UR) & MetSO (UR)**	0.64	0.11 – 0.89	0.025	moderate

LHP: lithium heparin plasma, SE: serum, UR: urine.
